# Trait Emotional Intelligence and School Burnout: The Mediating Role of Resilience and Academic Anxiety in High School

**DOI:** 10.3390/ijerph17093058

**Published:** 2020-04-28

**Authors:** Caterina Fiorilli, Eleonora Farina, Ilaria Buonomo, Sebastiano Costa, Luciano Romano, Rosalba Larcan, Konstantinos V. Petrides

**Affiliations:** 1 Department of Human Sciences, University of Rome, LUMSA, 00193 Rome, Italy; 2 Department of Human Sciences, University of Milan, 20126 Bicocca, Italy; 3 Department of Psychology, University of Campania Luigi Vanvitelli, 81100 Caserta, Italy; 4 Department of Clinical and Experimental Medicine, University of Messina, 98100 Messina, Italy; 5 London Psychometric Laboratory, Department of Clinical, Educational, and Health Psychology, University College London, London WC1H 0AP, UK

**Keywords:** trait emotional intelligence, school burnout, anxiety, resilience, adolescents, TEIQue

## Abstract

The main aim of the current study was to investigate the role of trait emotional intelligence (TEI) in preventing students’ school burnout directly and indirectly via anxiety and academic resilience. The data were derived from a sample of 1235 high school students (962 females and 273 males), ranging in age between 13 and 17 years (mean = 15.46; stand deviation = 1.22). Structural equation modelling revealed a strong indirect effect of TEI on school burnout, mediated via anxiety and resilience. Overall, students with high TEI were less likely to experience school anxiety and more likely to exhibit resilience which, in turn, reduced school burnout risk. Findings are discussed with reference to the wider role of TEI in educational contexts and highlight the need and potential for scientifically driven interventions to enhance emotional adjustment at school and in life, more generally.

## 1. Introduction

Recent investigations on adolescents’ risk of school dropout have shown a high rate of burned out students [1,2]. In this regard, an underestimation of the prevalence and impact of school burnout amongst high school students can hinder or impair their academic career and possibly also their well-being. Like other studies in the literature (e.g., [3] for a review), previous results suggest that trait emotional intelligence (TEI) has a strong impact, direct as well as indirect, on the affect-laden aspects of school burnout, anxiety, and resilience, which are undoubtedly very substantial. 

Trait emotional intelligence (TEI), also labeled trait emotional self-efficacy, is a set of emotion-related personality dispositions concerning how people manage emotions and understand the impact of their emotions on social interactions [4]. Core dimensions of TEI include people’s perceived ability to understand and regulate their emotions and to cope with stressful and emotional challenges occurring in their life. Such dimensions refer to both people’s own emotional experience and their perception of others’ experiences. According to its first conceptualization, TEI is conceived of as a distinct component of people’s personality [3,5]. Subsequent studies have largely proven that TEI is an emotion-related self-perception distinguishable from cognitive and emotional abilities [6,7,8]. Rather, the construct involves dispositional variables deriving, for example, from childhood temperament [9,10], and genes [11]. Conversely, findings regarding the role of contextual variables on shaping children’s TEI, like parenting styles [12], are rather mixed. Several researchers have found that children’s TEI may impact on subsequent development of social and emotional issues (e.g., [13,14]). In this regard, individuals with low TEI are more likely to use maladaptive coping strategies when they face stressful situations [15]. Additionally, a low level of TEI is significantly associated with anxiety, anger, disruptive behaviors, and with both internalizing and externalizing symptoms [16]. In contrast, Mavroveli and colleagues [17] have found that adolescents with a high level of TEI are generally less predisposed to developing depressive or somatic symptoms, and are more likely to successfully cope with difficult events [18]. Moreover, previous research has found that people with high TEI show a positive attitude towards stressful events, namely resilience [19,20,21,22]. Further, research findings have supported the link between personal resilience and TEI [23]. Effectively, data in different ages, such as in childhood [24], in adolescence [25], as well as in adulthood [26], have confirmed that high TEI people are more likely to face challenges positively.

Focusing on adolescents’ school-life, some authors have found a positive and predictive association between students’ TEI and their level of school adjustment, in terms of pro-social behavior, cooperation, and friendship [27,28]. Nevertheless, to our knowledge, no previous study has analyzed the relationship between students’ TEI and how emotionally demanding they find school tasks which, in turn, may lead to burnout [29]. School burnout has been studied by Salmela-Aro and colleagues [30] in terms of three inter-related aspects: exhaustion due to school demands, cynical and detached approach toward school, and feelings of inadequacy as a student. Several studies have demonstrated that students’ exhaustion is comparable to an emotional erosion occurring when they feel underequipped to deal with school demands, which they perceive as overwhelming (for a review, [31]). Students with school burnout show low interest and motivation to pursue desirable goals, such as school attendance and achievement [2], as well as long-term ones, like academic and occupational aspirations [32]. Furthermore, findings from longitudinal studies have consistently supported the association between high school burnout and emotional issues, such as depression and anxiety symptoms [33,34]. In a similar vein, previous research has also found that students with a low level of school-related emotional exhaustion are more resilient and able to ‘bounce back’ from negative experiences [35,36].

Current literature shows that TEI is a set of personality dispositions centered on emotions, thus the construct is likely to show significant associations with school anxiety, resilience, and school burnout. Moreover, studies have repeatedly demonstrated that students’ levels of anxiety and resilience are associated with school burnout. Accordingly, we hypothesized that anxiety and resilience mediate the relationship between TEI and school burnout. More specifically, we expected that TEI will be associated positively with resilience and negatively with anxiety and school burnout; furthermore, we expected that school burnout will be associated positively with anxiety and negatively with resilience. Finally, we expected that TEI will directly predict anxiety and resilience and also exert through both of them a significant indirect effect on school burnout. For a more conservative model, student age was controlled for in the analyses. 

## 2. Methods

### 2.1. Participants

The present study used a cross-sectional descriptive design with a convenience sampling procedure. The participants were 1235 high school students (Female *n* = 962, Male *n* = 273), ranging in age between 13 and 17 years (mean (*M*) = 15.46; standard deviation (*SD*) = 1.22). All students were Italian speaking and came from the three main geographical areas of Italy (north, center, and south of Italy), equally distributed. The research protocol was approved by the Ethics Committee of the Lumsa University of Rome, Italy.

### 2.2. Instruments

Trait Emotional Intelligence. Trait emotional intelligence was evaluated by the Trait Emotional Intelligence Questionnaire-Short Form for adolescents (TEIQue-ASF; [37]; Italian adaptation by [38]). The TEIQue-ASF is a self-report questionnaire composed of 30 items based on a 7-point Likert scale rating ranging from 1 (completely disagree) to 7 (completely agree). An example item is: “I find it hard to know exactly what emotion I’m feeling.” In this study, Cronbach’ s alpha for the total score was 0.79.

School anxiety and resilience. The Italian Questionnaire for Anxiety and Resilience for students (QAR; [39]) was used to evaluate school anxiety and resilience. The QAR is composed of 14 items based on a 5-point Likert scale (1 = Not at all, 5 = Totally). It measures two separate dimensions: anxiety and resilience. An example item of the anxiety dimension is: “The closer the date of an exam/verification in class, the more anxious I get.” An example item of the resilience dimension is: “I manage to overcome my disappointment for academic failure.” In the present study, Cronbach’s alpha were 0.87 for the anxiety subscale and 0.62 for the resilience subscale.

Students’ burnout. Students’ burnout was evaluated by the Italian version of School Burnout Inventory (SBI; [40]). The SBI is composed of nine items based on a 6-point Likert scale (1 = “I totally disagree”, 6 = “I totally agree”). It has been largely used as a unidimensional measure with good psychometric properties [30]. An example item is: “I feel like I’m losing interest in school.” In this study, Cronbach’s alpha for the total score was 0.85.

### 2.3. Data Analysis

Correlations and descriptive analyses were conducted for all variables in the study. To test the hypothesized model, Structural Equation Model (SEM) with maximum likelihood estimation and a 5000 resample of bootstrapped estimates was used to test mediation in line with Preacher and Hayes [41]. School burnout was represented by the three subscales of this measure (exhaustion, detachment, and inadequacy). The indicators of the latent variables for TEI, anxiety, and resilience were represented by three composite scores (parcels), averaging the respective scale item scores.

### 2.4. Procedure

The authors assert that all procedures contributing to this work comply with the ethical standards of the relevant National and Institutional Committees on Human experimentation. Students completed the questionnaires at school in a single session, lasting approximately 20 min. During the administrations, a member of the research team was present in case of need. Anonymity standards were assured for all participants. Teachers were not allowed to stay in class during the data collection, and students were assured that no one except the researchers would have access to their responses. Participation was voluntary, all required guarantees for privacy were extended and all students provided a signed consent form from their parents before participating in the research, following the ethical standards of the Declaration of Helsinki of 1964, article 20, 21, and 24, and its latest version. 

## 3. Results

Mean scores (M), standard deviations (SD), skewness, kurtosis, and zero-order correlations for all the study variables are displayed in Table 1. Correlations were statistically significant and in line with theoretical expectations. Thus, TEI was positively related to resilience, and negatively related to anxiety and school burnout. There was a strong negative correlation between school burnout and resilience, as well as a positive correlation between school burnout and anxiety. Age was positively correlated with school burnout while unrelated to TEI.

**Figure 1:**
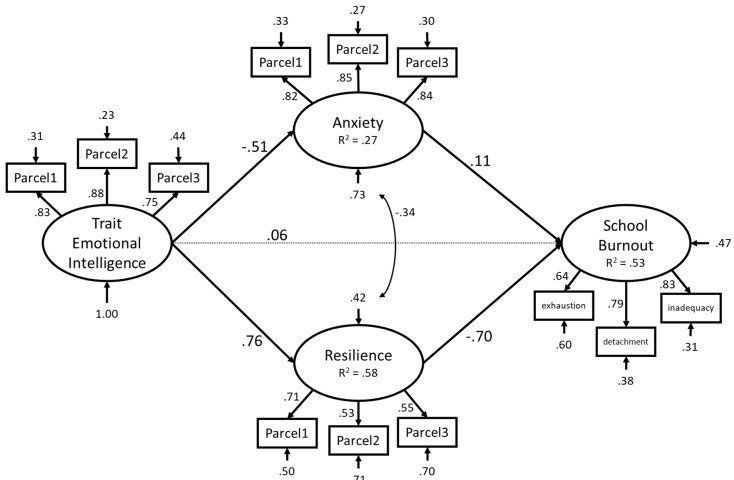
Structural equation model interlinking trait emotional intelligence (TEI), anxiety, resilience, and school burnout. Coefficients shown are standardized path coefficients. Parcels represent aggregation of items used as indicators of latent constructs. Paths from age are omitted for presentation purposes and are: age–TEI: *β* = 0.01; age–anxiety: *β* = −0.07; age–resilience: *β* = −0.09; age–school burnout: *β* = 0.10).

Structural equation modelling (SEM) with latent variables was used to test a model comprising the following paths: Anxiety and resilience as predicted by TEI, and school burnout as predicted by TEI, anxiety, and resilience. The effect of age in the model was controlled by linking it to all other variables in the model (TEI, anxiety, resilience, and school burnout). This model is depicted in Figure 1 (without age for simplicity).

The model showed a good fit to the data, *χ2* (56) = 375.62, *p* < 0.01; Comparative fit index (CFI) = 0.95, Standardised Root Mean square Residual (SRMR) = 0.05, Root Mean Square Error of Approximation (RMSEA) = 0.07 (90% confidence interval (CI) = 0.06; 0.08). The results of direct paths indicated that TEI directly predicts resilience (*b* = 0.62, 95% CI: (0.56; 0.68), β = 0.76, *p* < 0.001) positively, and anxiety (*b* = −0.57, 95% CI: (−0.64; −0.50), *β* = −0.51, *p* < 0.001) negatively, but not school burnout (*b* = 0.25, 95% CI: (−0.30; 0.92), *β* = 0.06, *p* = 0.43). Anxiety (*b* = 0.38, 95% CI: (0.02; 0.73), *β* = 0.11, *p* = 0.04) and resilience (*b* = −3.39, 95% CI: (−4.55; −2.51), *β* = −0.70, *p* < 0.001), in turn, predicted school burnout (positively and negatively, respectively). Age was also a significant predictor, positively predicting school burnout (*b* = 0.22, 95% CI: (0.08; 0.35), *β* = 0.10, *p* = 0.002), while negatively predicting anxiety (*b* = −0.05, 95% CI: (−0.08; −0.01), *β* = −0.07, *p* = 0.01) and resilience (*b* = −0.04, 95% CI: (−0.07; −0.02), *β* = −0.09, *p* = 0.002). Age was not significantly associated with TEI (*b* = 0.01, 95% CI: (−0.03; 0.04), *β* = 0.01, *p* = 0.76). Notably, the total indirect effect from TEI to school burnout was significant (*b* = −2.32, 95% CI: (−3.04; −1.79), *β* = −0.58, *p* < 0.001) comprising two subcomponents; one via anxiety (*b* = −2.10, 95% CI: (−2.86; −1.54), *β* = −0.53, *p* < 0.001) and the other via resilience (*b* = −0.22, 95% CI: (−0.42; −0.01), *β* = −0.05, *p* = 0.04). This ensured that the total effect of TEI on school burnout reached statistical significance (*b* = −2.07, 95% CI: (−2.41; −1.77), *β* = −0.52, *p* < 0.001).

## 4. Discussion

The current study investigated the interrelationships between TEI, academic anxiety, resilience, and school burnout level on a large sample of high school students. The core focus was on whether, and to what extent, students’ TEI predicted their school burnout levels, via school anxiety and resilience. The possible effect of student age was controlled for in the model. Overall, the findings supported both the correlational and the mediational hypotheses.

There was a negative correlation between students’ TEI and their school burnout levels, as expected. A closer look at the two constructs highlights their conceptual overlap in emotional content. TEI comprises important dimensions such as well-being, self-control, emotionality, and sociability, which might be expected to correlate with such aspects of students’ school life such as emotional exhaustion, alienation, and detachment from school events, and a sense of inadequacy in facing challenging school tasks. Our results show that students who are higher on trait emotional self-efficacy are less likely to feel overwhelmed by school tasks, which echoes previous findings in the literature (e.g., [28,42,43]). Furthermore, the associations between students’ TEI, academic anxiety and resilience were also consistent with existing literature from various countries around the world [16,17,20,44,45]. Similarly and in line with the existing literature, the correlational pattern emerging in this study revealed an untoward nexus of associations wherein students with high levels of burnout tended to feel less resilient and more anxious and doubtful about their emotional abilities [46,47].

In accordance with the mediation hypothesis, the results revealed a strong indirect predictive role of TEI on school burnout, via a dual route of anxiety and resilience. Overall, students with high TEI levels were less likely to experience school anxiety and more likely to exhibit resilience, which in turn reduced school burnout risk. In other words, positive emotional perceptions are a barrier to the experiencing of school burnout in a process that is mediated via low anxiety and high resilience. These results are in line with existing literature (partly reviewed in Petrides, Sanchez-Ruiz, Siegling, Saklofske, and Mavroveli [7]) showing that lower TEI students are more likely to experience anxiety when dealing with challenging school events, which in turn enhances their overall risk of school burnout [48]. Other scholars have posited that constructs related to emotional intelligence can prevent maladjustment and perceived stress through enhancing resilience in the academic context [48,49,50,51]. When students are able to count on personal resources, such as resilience, they are in a much better position to overcome acute or chronic adversities that can compromise their academic career [52,53,54,55]. 

Specifically, in relation to academic anxiety, previous research has found that it is strongly predicted by students’ self-regulation processes, which tend to be activated at the perception of challenging events [56]. Students with high anxiety levels are inclined to express worse evaluations of their social interactions compared to their low anxiety peers [57,58]. Overall, the results of this study support the status of trait emotional intelligence as a fundamental antecedent variable with a strong impact on students’ school adjustment outcomes [24,59].

Students’ age had rather limited effects in our analyses, likely as a result of its limited range (from 13 to 17 years) in our sample. Consistent with the existing literature (e.g., [30,40]), age was positively linked to school burnout levels, indicating that students are more likely to experience burnout as they progress into the higher and more challenging levels of secondary education. With respect to the lack of a relationship between age and TEI, the limited age-range of the sample may well have played a decisive role, since it does not allow for an examination of underlying trends from childhood into young adulthood (for a review, [7]; see also [60]). 

This study has a number of limitations that future research could try to remedy. First, a more balanced (with respect to gender) sample may allow for gender-specific analyses of effects to establish if gender acts as a moderator of some of the identified relationships. Second, it would be informative to complement self-report measures with data from other sources, such as peer- and teacher-reports (e.g., [13]). Third, a longitudinal expansion of the research design into more age groups could provide clarifications and insights not only in relation to the somewhat inconsistent function of age in the literature, but also in relation to the causal direction of the observed effects and interrelationships.

## 5. Conclusions

Underestimating the prevalence and impact of school burnout amongst high school students can hinder or impair their academic career and possibly also their well-being. The current study suggests that TEI has a strong influence, direct as well as indirect, on the affect-laden aspects of school burnout, anxiety, and resilience, which are undoubtedly very substantial. Future intervention programs in schools should be focused on the optimization of TEI levels, which will have a beneficial effect on a range of important outcomes in students’ daily life. Taken together, the present findings highlight the multifaceted role of emotional perceptions in the school environment and the need for those involved in educational policy and delivery to familiarize themselves with the theory and practice of trait emotional intelligence.

## Figures and Tables

**Table 1:** Descriptive statistics and intercorrelations between the variables in the study.

Variables	Min	Max	M	SD	Skew	Kurt	1	2	3	4
1. TEI	2.00	6.43	4.52	0.70	−0.06	−0.02	α = 0.82			
2. Anxiety	7.00	35.00	20.34	6.77	0.07	−0.84	−0.44 **	α = 0.86		
3. Resilience	7.00	35.00	21.76	4.43	0.06	−0.06	0.58 **	−0.42 **	α = 0.61	
4. School burnout	9.00	54.00	29.85	9.05	−0.01	−0.45	−0.45 **	0.45 **	−0.53 **	α = 0.84
5. Age	13.00	17.00	15.46	1.22	−0.01	−1.48	0.01	−0.07 **	−0.08 **	0.12 **

Note: M, Mean; SD, standard deviation; TEI, trait emotional intelligence. *N* = 1235; ** *p* < 0.01.
